# Longitudinal Associations Between Inflammatory Markers at 12‐Months and Three Core Symptoms of Depression at 12‐ and 24‐Months After Colorectal Cancer Diagnosis: Results From the Population‐Based PROFILES Registry

**DOI:** 10.1002/pon.70555

**Published:** 2026-07-18

**Authors:** Chris Hinnen, Floortje Mols, Dounya Schoormans

**Affiliations:** ^1^ Department of Medical Psychology Spaarne Gasthuis Haarlem the Netherlands; ^2^ CoRPS—Center of Research on Psychological Disorders and Somatic Diseases Department of Medical and Clinical Psychology Tilburg University Tilburg the Netherlands; ^3^ Department of Research & Development Netherlands Comprehensive Cancer Organisation (IKNL) Utrecht the Netherlands

**Keywords:** cancer, colorectal, depression, inflammation, oncology, PROFILES

## Abstract

**Purpose:**

Depression is a heterogeneous construct comprising distinct symptom domains, including motivational anhedonia, consummatory anhedonia, and negative affect, commonly experienced by colorectal cancer (CRC) survivors. Inflammation has been implicated in depression, its association with specific depressive symptom domains in CRC survivors remains insufficiently characterized.

**Methods:**

CRC patients (*n* = 497) completed questionnaires assessing depressive symptoms 12‐ and 24‐months post‐diagnosis: motivational anhedonia (Multidimensional Fatigue Inventory), consummatory anhedonia (Hospital Anxiety and Depression Scale–depression), and negative affect (EORTC QLQ‐C30, emotional functioning). Associations between 11 inflammatory markers (CRP, IL‐1α, IL‐1β, IL‐6, IL‐8, IL‐10, IL‐17A, IL‐22, IFN‐γ, sTNFRI, and sTNFRII) at 12‐months and depressive symptoms at 12‐ and 24‐months were examined using linear mixed models.

**Results:**

IL‐1β (Est = 0.089, *p* = 0.003) and IFN‐γ (Est = 0.067, *p* = 0.001) were associated with more consummatory anhedonia symptoms across time, and a small sTNFRI × time interaction was found (Est = 0.000, *p* = 0.007). Negative affect was associated with IL‐1β (Est = −0.496, *p* < 0.001), IFN‐γ (Est = −0.354, *p* < 0.001), and sTNFRI (Est = −0.003, *p* < 0.001). A significant IL‐1*α* × time interaction was observed (Est = 0.250, *p* = 0.038) for motivational anhedonia.

**Conclusions:**

Most consistently IL‐1β, IFN‐γ, and sTNFRI was associated with greater consummatory anhedonia and more negative affect in CRC survivors. In addition, inflammatory correlates (IL‐1α and sTNFRI) of motivational anhedonia and consummatory anhedonia changed over time. These findings suggest that selected inflammatory pathways may contribute to specific depressive symptom domains during colorectal cancer survivorship. Given the exploratory nature of the analyses, replication in independent cohorts is required before clinical implications can be drawn.

## 1 Introduction

Depression is highly prevalent in patients with colorectal cancer (CRC) [1]. According to the DSM‐5, depression is characterized by three core symptom domains: (1) lack of interest (motivational anhedonia), (2) lack of pleasure or positive affect (consummatory anhedonia), and (3) the presence of negative affect (e.g., sadness, worry, despair) [2]. Recent studies have supported this distinction in both cancer patients in general [3] and CRC patients specifically [4]. Although still uncommon in oncology research, increasing attention is being paid to examining depressive symptoms individually rather than treating depression as a unitary syndrome [5, 6]. We previously demonstrated among CRC patients that trajectories of depressive domains depend on illness phase, with high levels of all three domains at diagnosis, which recover after 12‐months and remain largely stable in the year thereafter [4]. This is in line with other studies describing (domains of) depressive symptoms to recover after the first 12‐months after diagnosis/acute treatment phase [7, 8] We specifically focused on these three domains because they represent the core symptom dimensions that define depressive disorders according to DSM‐5 diagnostic criteria. Increasing evidence indicates that these symptom domains differ in their biological correlates and longitudinal trajectories, suggesting that investigating them separately may provide greater mechanistic insight than studying depression as a single homogeneous construct.

These different depressive symptoms may have distinct aetiologies and neurobiological underpinnings and may therefore require tailored treatment approaches. Inflammation, particularly elevated levels of pro‐inflammatory cytokines, has been implicated as a potential contributor to depressive symptoms [9]. Indeed, a growing body of evidence supports associations between inflammation and depressive symptoms, both in the general population [10] and among cancer patients [9]. Although most oncology studies have not differentiated between core depressive symptom domains in relation to inflammation, research in the general population suggests that anhedonia (i.e., lack of interest and/or pleasure), rather than negative affect, may be most strongly associated with inflammation [11, 12]. One possible explanation is that inflammation induces anhedonia (i.e., lack of interest and pleasure) as part of ‘sickness behaviour’ (e.g., social withdrawal, passivity [13]), which may have evolved to promote survival during periods of illness. Understanding the relation between inflammation and differentiated core depressive domains among cancer patients is important, as it may help to identify patients at risk of specific elevated depressive symptoms and guide the development of tailored interventions. Although these three symptom domains are conceptually derived from the DSM‐5, no single questionnaire was specifically developed to measure motivational anhedonia, consummatory anhedonia, and negative affect as separate constructs in oncology populations. Therefore, the present study operationalized these domains using validated symptom scales that most closely represent their underlying psychological constructs. Throughout this manuscript, the terms motivational anhedonia, consummatory anhedonia, and negative affect therefore refer to operationalized symptom domains rather than pure diagnostic entities.

A specific understanding of the association between inflammation and depressive symptom domains during cancer survivorship is lacking, while this is important as depressive symptoms and acute inflammation have typically stabilized beyond the acute cancer(treatment) phase [4, 14]. In the current study we will therefore examine this association over a period of 12–24 months after diagnosis as this enables assessment of the association between inflammation and core depressive symptom domains beyond the acute (treatment) phase. The present study aimed to (1) examine the level and course of the three core symptoms of depression (i.e., motivational anhedonia, consummatory anhedonia and negative affect) from 12 months post diagnosis till 24 months after diagnosis; and (2) investigate the associations between inflammation at 12 months and these three core symptoms of depression at 12‐ and 24‐ months after diagnosis. Based on prior research, we hypothesized that pro‐inflammatory cytokines would show a stronger positive association with anhedonia, yet less with negative affect, between 12‐ and 24‐months post‐diagnosis. Associations are examined while taken clinical factors into account, that is, comorbidities (e.g., heart disease, high blood pressure, asthma, diabetes), tumor type, stage, and type of treatment (chemo‐ and/or radiotherapy as they are known to cause inflammation [15, 16]).

## 2 Methods

### 2.1 Setting and Participants

Data were derived from PROCORE, a prospective population‐based study designed to evaluate the impact of CRC and its treatment on patient‐reported outcomes [17]. Between January 2016 and January 2019, adult patients newly diagnosed with CRC recruited in four Dutch hospitals (Elisabeth‐TweeSteden hospital, Catharina hospital, Elkerliek hospital, and Máxima Medical Centre). Individuals were excluded if they had a history of malignancy other than non‐melanoma skin cancer, suffered from severe cognitive limitations, or were unable to complete questionnaires in Dutch. The PROCORE received ethical approval from the certified Medical Ethic Committee of Medical research Ethics Committees United (registration number: NL51119.060.14) and was conducted according to the declaration of Helsinki.

Details regarding data collection have been published elsewhere [17]. In short, data were collected via the PROFILES registry, which monitors the physical and psychosocial impact of cancer(treatment) [18]. PROFILES is linked to the Netherlands Cancer Registry (NCR), which systematically captures clinical data on all newly diagnosed cancer cases nationwide [19]. Eligible patients were approached in the hospital setting by their case manager or research nurse shortly after diagnosis and prior to initiating treatment. A small number of patients were inadvertently enrolled despite previous cancer diagnosis or having already commenced treatment. Consistent with earlier PROCORE publications [20], these patients were excluded from analyses.

### 2.2 Data Collection

Eligible patients received a study package containing written information about PROCORE, including an information letter, informed consent form, baseline questionnaire, and materials required for blood collection. Patients provided these materials to their hospital, where their blood was drawn. Written informed consent was obtained prior to patient participation Assessments were conducted at diagnosis (prior to treatment), 12 and 24 months post‐diagnosis. The present analyses specifically focused on inflammatory biomarkers obtained 12 months after diagnosis, when the majority of patients had completed primary (curative) treatment. Consequently, the analyses primarily reflect the survivorship phase rather than acute treatment‐related inflammatory responses.

#### 2.2.1 Inflammatory Markers

Blood samples were processed according to a harmonized standard operating procedure (SOP) previously implemented across participating hospitals [21]. After centrifugation, plasma fractions were temporarily stored locally at −80°C until further handling. Subsequently, samples were transported on dry ice (−80°C) to a centralized biobank facility. Samples were pseudo‐anonymized using a coded identifier consisting of study‐ID, hospital site, participant number, and sample type (i.e., plasma, buffy coat, or whole blood).

Based on prior literature regarding inflammatory processes in cancer‐related outcomes, eleven biomarkers were selected for measurement. For each participant, one plasma aliquot was thawed for immediate analysis, while additional aliquots were preserved to support future investigations. A panel of 11 inflammation‐associated biomarkers was selected for quantification in undiluted serum samples: C‐reactive protein (CRP) and the following cytokines: Interleukin‐1 alpha (IL‐1α), IL‐1 beta (IL‐1β), IL‐6, IL‐8, IL‐10, IL‐17A, IL‐22, Interferon gamma (IFN‐γ); and proxies for Tumor Necrosis Factor‐alpha (TNF‐alpha) soluble Tumor Necrosis Receptors 1 and 2 (sTNFRI and sTNFRII). Concentrations were determined in duplicate using a customized bead‐based multiplex immunoassay (ThermoFisher, USA) analyzed on the Luminex 200 platform (xMAP Technology, the USA), in accordance with the manufacturer's instructions and [22, 23].

### 2.3 Questionnaires

#### 2.3.1 Sociodemographic and Clinical Characteristics

Sociodemographic (i.e., sex, age) and clinical characteristics (i.e., comorbidities, cancer type, clinical stage, treatment) information were obtained from the NCR [19]. Socio‐Economic Position (SEP) was based on standardized household income from Dutch postal codes (four digits and two letters), provided by the Netherlands Institute of Social Research. Decile scores were grouped as low (1–3), medium (4–6) and high (7–9) SEP. Number of co‐morbidities (i.e., asthma/COPD, diabetes mellitus, heart disease, hypertension, osteoarthritis, rheumatoid arthritis, stroke, stomach, kidney, liver, or thyroid disease) were assessed using an adapted version of the Self‐administered Comorbidity Questionnaire (SCQ) [24] and recoded into none, one, and two or more.

#### 2.3.2 Depressive Symptoms

*Motivational anhedonia* was operationalized using the four‐item Reduced Motivation subscale of the Multidimensional Fatigue Inventory (MFI) [25]. Although originally developed to assess reduced motivation within the multidimensional fatigue construct, this subscale reflects diminished initiative and reduced goal‐directed behaviour, characteristics that conceptually overlap with motivational anhedonia. We therefore considered this instrument an appropriate proxy for motivational anhedonia while acknowledging that dedicated reward‐processing instruments may capture this construct more specifically. Items are scored on a five‐point Likert scale and were reverse‐coded so that higher values reflect lower motivation. The MFI has demonstrated validity in oncology populations [26], and the motivation subscale shows moderate associations with depressive and anxiety symptoms in cancer patients [27]. Internal consistency in the present sample was acceptable (*α* = 0.75 at 12 months; *α* = 0.77 at 24 months).

*Consummatory anhedonia* was operationalized using the depression subscale of the Hospital Anxiety and Depression Scale (HADS‐D) [28, 29]. Although the HADS‐D is commonly interpreted as a measure of depressive symptom severity, psychometric studies have shown that its depression items primarily assess diminished enjoyment, reduced pleasure, and loss of positive affect while deliberately minimizing somatic symptoms. Consequently, we considered the HADS‐D an appropriate pragmatic proxy for consummatory anhedonia in an oncology population, as it is widely interpreted as capturing reduced capacity for pleasure [30, 31]. Each item is rated on a four‐point scale, with total scores ranging from 0 to 21. Reliability coefficients were 0.82 (12 months) and 0.85 (24 months).

*Negative affect* was assessed using the four‐item emotional functioning subscale of the EORTC QLQ‐C30 (version 3.0) [31]. Scores were linearly transformed to a 0–100 scale, with lower scores indicating greater negative affect. Cronbach's alpha was 0.89 at 12 months and 0.87 at 24 months.

### 2.4 Statistics

Descriptive statistics were calculated for sociodemographic (i.e., sex, age, SEP) and clinical characteristics (i.e., tumor type, stage, type of treatment), depressive symptoms, and inflammatory markers. Continuous variables were reported as mean ± standard deviation (SD) and categorical variables as numbers and frequencies. Given the skewness of inflammatory markers, log‐transformations were applied prior to statistical analyses. Biomarker values below assay detection limits were considered as missing and imputed using multiple imputation (1000 iterations), constrained between zero and the assay‐spesific lowest detection threshold. Potential outliers were inspected visually; as all values were within the predefined threshold of 1.5 times the interquartile range, none were excluded. Values deemed implausible were recoded as missing.

Longitudinal associations were examined using linear mixed models with maximum likelihood estimation and an unstructured covariance matrix, specifying a two‐level structure (time level and patient level). This approach accommodates varying numbers of observations per assessment; therefore, missing data were not imputed. To examine the level and course of the three core symptoms of depression (i.e., motivational anhedonia, consummatory anhedonia and negative affect) from 12‐months post diagnosis till 24 months after diagnosis, mixed models with no explanatory variables, only the intercept were calculated (i.e., an unconditional model). This analysis allowed for the determination of the amount of variance at the person and time level, for each core symptom separately. Second, time as explanatory variable (i.e., unconditional growth model) was calculated to determine whether motivational anhedonia, consummatory anhedonia and negative affect differs between 12 and 24 months.

To assess whether association between inflammation at 12‐months and the three core symptoms of depression at 12‐ and 24‐months after diagnosis, the 11 inflammatory markers were added separately to the models together with sociodemographic and clinical variables to control for their effects. Interaction terms (inflammation × time) were added to test whether 12‐month inflammation was associated with differential symptoms trajectories. Time served two purposes within the mixed models. First, it allowed us to examine whether depressive symptom levels changed between 12 and 24 months after diagnosis. Second, interaction terms between each inflammatory marker and time were included to determine whether inflammatory activity measured at 12 months predicted changes in depressive symptom trajectories over follow‐up rather than only average symptom levels.

Given the exploratory nature of this new study, we applied a conventional two‐sided significance level of *p* ≤ 0.05 and did not correct for multiple testing, to avoid overlooking potentially meaningful patterns that warrant further investigation. Analyses were run using SPSS (version 24).

## 3 Results

### 3.1 Sensitivity Analyses of Potential Pre‐Analytical Influences

Prior to the primary analyses, we evaluated whether transient pre‐analytical factors that could potentially influence inflammatory biomarker concentrations (including caffeine consumption, influenza vaccination, and physical activity) should be considered as additional covariates. Variables showing initial associations with biomarker concentrations were subsequently evaluated in sensitivity analyses. Because these associations disappeared after excluding a small number of influential observations, these factors were not retained in the primary analytical models.

### 3.2 Sociodemographic and Clinical Characteristics

Descriptive analyses for sociodemographic and clinical characteristics of the study sample are presented in Table 1. A total of 497 patients were included, of whom 291 (60.8%) were male. Most patients were diagnosed with colon cancer (70.8%) and had stage‐III disease (37.8%). All patients underwent surgery, and the majority (61%) did not receive adjuvant treatment.

**TABLE 1:** Sample characteristics.

Depression questionnaires completed	12 or 24 months (*N* = 479)	12 and 24 months (*N* = 311)	12 not 24 months (*N* = 46)
Age, mean (SD)	67.08 (9.23)	66.5 (8.70)	68.11 (8.91)
Sex, total (%)			
Male	291 (60.8%)	193 (62,1%)	28 (60.9%)
Female	188 (39.2%)	118 (37.9%)	18 (39.1%)
SEP, total (%)			
Low	126 (26.5%)	85 (27,3%)	12 (26.1%)
Middle	231 (48.6%)	152 (48.9%)	20 (43.5%)
High	118 (24.8%)	722 (3.2%)	13 (28.3%)
Co‐morbidity, mean (SD)	1.94 (1.66)	1.87 (1.63%)	2.65 (1.97)
Type of cancer, total (%)			
Colon	339 (70.8%)	225 (72.3%)	27 (58.7%)
Rectum	140 (29.2%)	86 (27.7%)	17 (37.0%)
Stage, total (%)			
I	139 (29%)	106 (34.1%)	8 (17.4%)
II	139 (29%)	85 (27.3%)	13 (28.3%)
III	181 (37.8%)	114 (36.7%)	20 (43.5%)
IV	19 (4%)	5 (1.6%)	5 (10.9%)
unknown	1 (0.2%)	1 (0.3%)	
Adjuvant treatment, total (%)			
No adjuvant treatment	292 (61%)	198 (63.7%)	24 (52.2%)
Chemotherapy	102 (21.3%)	70 (22.5%)	9 (19.6%)
Radiation therapy	32 (6.7%)	20 (6.4%)	3 (6.5%)
Chemo and radiation therapy	53 (11.1%)	23 (7.4%)	10 (21.7%)

*Note: N*=Numbers (% = percentages) and age in mean years (SD).

Abbreviations: SD = standard deviation; SEP = social economic position.

### 3.3 Depressive Symptoms and Inflammatory Markers

Table 2 presents mean (SD) scores for inflammatory markers at 12‐months and depressive symptoms at 12‐ and 24‐months. At group levels depressive symptoms appeared stable between both measurement occasions.

**TABLE 2:** Mean scores on depressive symptoms and inflammation markers at 12‐ and 24‐months after diagnosis.

	12 months	24 months
Depressive symptoms, mean (SD)		
Motivational anhedonia (MFI—RM)	8.55 (3.69)	8.72 (3.75)
Consummatory anhedonia (HADS—D)	3.43 (3.31)	3.97 (3.69)
Negative affect (EORTC QLQ‐C30—EF)	89.74 (17.44)	88.99 (16.02)
Inflammatory markers, mean (SD)		
CRP	1.08 (1.61)	—
IL‐1α	0.27 (1.65)	—
IL‐1β	2.94 (7.31)	—
IL‐6	2.44 (26.36)	—
IL‐8	1.19 (8.71)	—
IL‐10	2.18 (3.27)	—
IL‐17A	2.92 (10.77)	—
IL‐22	8.42 (84.73)	—
IFN‐γ	17.57 (9.44)	—
sTNFRI	1628.16 (1263.26)	—
sTNFRII	45.39 (28.92)	—

*Note:* Numbers are presented in means with standard deviation (SD). MFI‐RM = Multidimensional Fatigue Inventory—Reduced Motivation subscale.

Abbreviations: CRP = C‐reactive protein; EORTC QLQ‐C30—EF, EORTC quality of life questionnaire C30, Emotional Functioning subscale; HADS‐D = Hospital and Anxiety Scale—Depression subscale; IFN‐*γ* = Interferon gamma; IL = interleukin; sTNFRI = soluble tumor necrosis factor receptor‐I; sTNFRII = soluble tumor necrosis factor receptor‐II.

#### 3.3.1 Motivational Anhedonia

Intraclass correlation coefficients (ICCs) from the unconditional model indicated that 65% of the variance was attributable to between‐person differences, with the remaining 35% reflecting within‐person fluctuations over time. Next, we entered Time into the model. Time was not significantly associated with motivational anhedonia (Est = −0.18, SE = 0.17, *p* = 0.29), indicating no difference in motivational anhedonia scores between 12‐ and 24‐months. In the next step, we added each inflammatory marker separately to the model. These analyses showed that none of the inflammatory markers were associated with motivational anhedonia, averaged across both time points, while taking clinical and sociodemographic factors into account (See Table 3). Where the model without an interaction term showed no significant average association with IL‐α, in the model including a time × IL‐α interaction (Table 4), the interaction term was significant (Est = 0.250, SE = 0.119, *p* = 0.038).

**TABLE 3:** Association between inflammatory markers and core depressive symptoms: Results from Linear mixed models adjusted for clinical and sociodemographic covariates.

	Motivational anhedonia			Consummatory anhedonia			Negative affect		
Inflammatory markers	Estimate (SE)	95% CI	*p*‐value	Estimate (SE)	95% CI	*p*‐value	Estimate (SE)	95% CI	*p*‐value
CRP	0.257 (0.192)	−0.121 to −0.635	0.182	0.228 (0.177)	−0.120 to −0.577	0.198	−0.139 (0.808)	−1.730 to −1.453	0.864
IL 1α	−0.046 (0.123)	−0.288 to −0.196	0.710	−0.066 (0.115)	−0.292 to −0.161	0.567	−0.141 (0.500)	−1.128 to −0.845	0.778
IL 1β	0.036 (0.032)	−0.027 to −0.100	0.262	**0.089 (0.029)**	**0.031 to −0.147**	**0.003**	**−0.496 (0.126)**	**−0.745 to −0.246**	**<** **0.001**
IL 6	−0.002 (0.006)	−0.015 to −0.010	0.716	−0.002 (0.006)	−0.014 to −0.010	0.765	0.002 (0.026)	−0.050 to −0.054	0.939
IL 8	−0.007 (0.019)	−0.046 to −0.031	0.705	−0.005 (0.018)	−0.041 to −0.031	0.785	0.014 (0.079)	−0.143 to −0.171	0.859
IL 10	−0.075 (0.075)	−0.223 to −0.073	0.321	0.029 (0.070)	−0.110 to −0.168	0.678	−0.460 (0.305)	−1.062 to −0.142	0.13
IL 17A	−0.027 (0.034)	−0.093 to −0.040	0.427	−0.035 (0.031)	−0.097 to −0.027	0.265	0.090 (0.137)	−0.180 to −0.360	0.511
IL 22	−0.005 (0.008)	−0.021 to −0.011	0.533	−0.003 (0.008)	−0.018 to −0.011	0.651	0.038 (0.033)	−0.026 to −0.103	0.244
IFN‐γ	0.034 (0.025)	−0.017 to −0.084	0.188	**0.067 (0.023)**	**0.030 to −0.122**	**0.001**	**−0.354 (0.101)**	**−0.553 to −0.155**	**<** **0.001**
sTNFRI	0.000 (0.000)	0.000 to −0.001	0.588	0.000 (0.000)	0.000 to −0.001	0.241	**−0.003 (0.001)**	**−0.005 to −0.001**	**<** **0.001**
sTNFRII	0.012 (0.009)	−0.004 to −0.029	0.145	0.006 (0.008)	−0.010 to −0.022	0.438	0.016 (0.035)	−0.053 to −0.085	0.648

*Note:* Models were adjusted for time, sociodemographic (age, gender, SES) and clinical variables (comorbidity, tumor type, stage, treatment). Significant values are denoted in bold.

Abbreviations: CRP = C‐reactive protein; IL = interleukin, IFN‐*γ* = Interferon gamma; sTNFRI = soluble tumor necrosis factor receptor‐I; sTNFRII = soluble tumor necrosis factor receptor‐II.

**TABLE 4:** Association between inflammatory markers and changes in core depressive symptoms *over time*: Results from linear mixed models adjusted for clinical and sociodemographic covariates.

Inflammatory markers	Changes in motivational anhedonia			Changes in consummatory anhedonia			Changes in negative affect		
	Estimate (SE)	95% CI	*p*‐value	Estimate (SE)	95% CI	*p*‐value	Estimate (SE)	95% CI	*p*‐value
CRP*time	0.162 (0.269)	−0.369 to −0.694	0.547	0.353 (0.219)	−0.079 to −0.784	0.108	1.222 (1.319)	−1.378 to −3.822	0.355
IL‐1α*time	**0.250 (0.119)**	**0.015 to −0.485**	**0.038**	−0.030 (0.097)	−0.222 to −0.162	0.758	−0.722 (0.603)	−1.913 to −0.469	0.233
IL‐1β*time	−0.042 (0.032)	−0.106 to −0.021	0.190	0.033 (0.026)	−0.019 to −0.084	0.211	−0.051 (0.162)	−0.372 to −0.270	0.753
IL‐6*time	−0.011 (0.006)	−0.023 to −0.002	0.098	−0.001 (0.005)	−0.011 to −0.009	0.820	−0.019 (0.032)	−0.083 to −0.045	0.559
IL‐8*time	−0.032 (0.019)	−0.070 to −0.006	0.102	0.000 (0.016)	−0.031 to −0.031	0.993	−0.059 (0.097)	−0.251 to −0.133	0.544
IL‐10*time	−0.036 (0.075)	−0.185 to −0.112	0.629	0.015 (0.061)	−0.105 to −0.135	0.806	−0.144 (0.377)	−0.889 to −0.601	0.703
IL‐17A*time	−0.030 (0.034)	−0.097 to −0.036	0.367	−0.013 (0.027)	−0.066 to −0.041	0.633	−0.140 (0.168)	−0.472 to −0.192	0.407
IL‐22*time	−0.005 (0.008)	−0.021 to −0.011	0.543	0.005 (0.006)	−0.008 to −0.017	0.455	−0.014 (0.040)	−0.093 to −0.065	0.724
IFN‐γ*time	−0.031 (0.025)	−0.082 to −0.019	0.220	0.025 (0.020)	−0.016 to −0.065	0.229	−0.022 (0.128)	−0.276 to −0.231	0.861
sTNFRI*time	0.000 (0.000)	−0.001 to −0.00	0.308	**0.000 (0.000)**	**0.000 to −0.001**	**0.007**	−0.000 (0.001)	−0.002 to −0.002	0.930
sTNFRII*time	0.004 (0.008)	−0.013 to −0.020	0.669	0.011 (0.007)	−0.002 to −0.024	0.104	0.016 (0.042)	−0.067 to −0.98	0.710

*Note:* Models were adjusted for time, sociodemographic (age, gender, SES) and clinical variables (comorbidity, tumor type, stage, treatment). Significant values are noted in bold.

#### 3.3.2 Consummatory Anhedonia

Interclass correlation coefficients of the unconditional model showed that 70% of the variance was attributable to differences between patients, and the remaining 30% represented fluctuations within persons over time. Next, we entered Time into the model. Time was significantly associated with consummatory anhedonia (Est = −0.59, SE = 0.15, *p* < 0.001). Patients scored lower at 12‐months (*M* = 3.43, SD = 3.31) compared to 24‐months (*M* = 3.97, SD = 3.69), mean difference = −0.59 (95% CI: −0.88 to −0.30, *p* < 0.001). In the next step, we added sociodemographic variables, clinical variables and inflammatory markers to the model. IL‐1β (Est = 0.089, SE = 0.029, *p* = 0.003) and IFN‐γ (Est = 0.067, SE = 0.023, *p* = 0.001) were positively associated with consummatory anhedonia over time (Table 3). sTNFRI was not significantly associated with consummatory anhedonia in models without an interaction term. After adding a time × sTNFRI interaction (Table 4), the interaction was significant (Est = 0.000, SE = 0.000, *p* < 0.01) with a very small effect, indicating that the sTNFRI—consummatory anhedonia association slightly differed across time.

#### 3.3.3 Negative Affect

Intraclass correlation coefficients indicated that 61% of the variance was attributable to between‐patient differences and 39% to within‐patient fluctuations over time. Next, we entered Time into the model. Time was not found to be significantly associated with negative affect indicating that negative affect at 12‐ and 24‐months did not differ. In the next step, we added inflammatory markers to the model. Analyses showed that IFN‐γ (Est = −0.354, SE = 0.101, *p* < 0.001), IL‐1β (Est = −0.496, SE = 0.126, *p* < 0.001), and sTNFRI (Est = −0.003, SE = 0.001, *p* < 0.001) were significantly associated with negative affect, after controlling for sociodemographic and clinical variables (See Table 3).

## 4 Discussion

This prospective study investigated associations between inflammatory markers and core depressive symptoms 12‐ to 24‐months after CRC diagnosis. Elevated levels of IL‐1β and IFN‐γ were associated with higher levels of consummatory anhedonia (i.e., lack of positive affect) and negative affect (i.e., feeling depressed, irritable, tense, worrying). Furthermore, sTNFRI (a proxy for TNF‐α [32]) was related to negative affect, whereas IL‐α and sTNFR1‐α showed time‐dependent associations with motivational and consummatory anhedonia, respectively, between 12‐ and 24‐months post‐diagnosis. This supports the notion that inflammation may relate differently to distinct depressive symptom dimensions across the course of survivorship. Overall, our findings reinforce accumulating evidence that systemic inflammation is linked to depressive symptoms in both community and oncological cohorts. Importantly, these associations were observed for only a subset of the investigated inflammatory markers, whereas several biomarkers frequently associated with depression in previous literature, including CRP and IL‐6, were not significantly associated with any of the examined symptom domains. This pattern likely reflects the complexity and redundancy of inflammatory signaling rather than evidence for a generalized inflammatory phenotype. Moreover, because multiple inflammatory markers were evaluated without adjustment for multiple testing, the possibility of chance findings cannot be excluded. Accordingly, the present findings should be regarded as exploratory and hypothesis‐generating until replicated in independent cohorts. However, most meta‐analyses have highlighted IL‐6 and CRP as the most consistent markers [9, 10]. This apparent discrepancy may partly reflect the historical emphasis on a relatively limited number of inflammatory biomarkers—particularly IL‐6, CRP, and TNF‐related signaling—in depression research. Consequently, these biomarkers have been investigated substantially more frequently than many other inflammatory mediators, potentially contributing to an overrepresentation of positive findings in the literature.

Our findings are consistent with literature, recent meta‐analyses confirm that peripheral concentrations of IL‐1β and TNF‐α are among the most robust inflammatory biomarkers associated with major depressive symptoms in clinical populations [33]. The biological plausibility of these associations is strengthened by mechanistic evidence showing that both cytokines activate p38 MAP‐kinase signaling, thereby up‐regulating the serotonin‐ and norepinephrine‐reuptake transporters and reducing synaptic monoamine availability [34]. Moreover, pro‐inflammatory cytokines, particularly IFN‐γ and TNF‐α, induce indoleamine‐2,3‐dioxygenase (IDO), which shunts the tryptophan pool away from serotonin synthesis towards the kynurenine pathway [34, 35]. Down‐stream metabolites of kynurenine, such as kynurenic and quinolinic acid, exhibit neuroactive and neurotoxic properties that can further contribute to mood disturbance [35]. Our observation that circulating IFN‐γ correlates with depressive symptoms is therefore in line with its dual capacity to (i) drive IDO‐mediated tryptophan depletion and (ii) interfere with dopaminergic neurotransmission—an effect also documented after therapeutic IFN‐α administration in humans and non‐human primates [36]. Taken together, these pathways provide a coherent biochemical framework explaining why IL‐1β, TNF‐α and IFN‐γ emerge as meaningful peripheral indicators of inflammation‐linked depression in our sample.

Interestingly, inflammation was not associated with motivational anhedonia, contrary to prior work linking inflammation to sickness behavior [35]. Instead, associations were observed only with affective components of depression (consummatory anhedonia and negative affect). This challenges recent hypotheses that link inflammation specifically to motivational features yet is consistent with a recent study that reported the involvement of inflammation in non‐sickness related behaviors [12]. This may be explained by timing of measurement: biomarkers were collected during survivorship (12–24 months post‐treatment), rather than during the acute treatment phase when sickness behavior is most pronounced.

### 4.1 Study Limitations

Several methodological considerations warrant cautious interpretation of our findings. First, the observational design precludes causal inference; although temporal ordering was respected, reverse causality and bidirectional effects between inflammation and depressive symptoms cannot be excluded. Second, inflammatory biomarkers were sampled at two post‐diagnosis time points (12‐ and 24‐months). Because we sampled biomarkers only twice, we may have missed brief cytokine spikes that occur during treatment or early survivorship, preventing us from fully mapping their trajectories or pinpointing periods of greatest vulnerability. Third, the depressive symptom domains were operationalized using validated self‐report questionnaires that represent clinically meaningful proxies rather than pure measures of motivational anhedonia, consummatory anhedonia, and negative affect. Although these instruments have demonstrated good psychometric performance in oncology populations, they inevitably capture overlapping aspects of depressive symptoms. Future studies should include dedicated reward‐processing instruments specifically developed to distinguish motivational and consummatory anhedonia. Consequently, our findings should be interpreted as reflecting operationalized symptom domains rather than the underlying DSM‐5 constructs themselves. Fourth, residual confounding is possible: variables such as concomitant medications (e.g., corticosteroids, antidepressants), physical activity, and sleep quality were not systematically recorded yet could influence both cytokine concentrations and mood. Finally, we analyzed each biomarker separately rather than as composite scores or network clusters. This approach may underestimate complex immune interactions or synergistic effects among cytokines and limits insight into broader immunological phenotypes (e.g., Th1 vs. Th2 balance). Collectively, these limitations highlight the need for future longitudinal studies with more frequent biomarker sampling, clinician‐administered psychiatric assessments, comprehensive behavioural covariates, and multivariate immune profiling to further elucidate the mechanistic links between inflammation and depression in cancer survivorship.

This study has several methodological strengths that enhance the credibility and relevance of its findings. First, the cohort comprised nearly 500 CRC patients recruited from multiple clinical centers, thereby enhancing statistical power and external validity. Second, instead of treating depression as a single score, we included various facets of depression, that is, motivational anhedonia, consummatory anhedonia, and negative affect, which provided a finer‐grained picture of mood disturbances. Third, all inflammatory markers were assayed centrally with validated, high‐sensitivity multiplex methods, and the panel included mediators rarely examined in previous oncology work, most notably soluble TNF receptors (sTNFR1/2) and IFN‐γ. Because sTNFR1 exhibits far greater serum stability than TNF‐α, its inclusion provided a more reliable index of TNF‐axis activation and may account for the robust associations detected in the absence of direct TNF‐α measurements. Fourth, our analytical strategy leveraged multilevel modelling across two post‐diagnosis time points, capturing both between‐patient heterogeneity and within‐patient change. Together, these strengths position the present study as a substantive contribution to the literature on inflammation‐linked mood disturbances in cancer survivorship.

### 4.2 Clinical Implications

The present findings carry several translational implications. Across depressive symptom domains, inflammatory activity at 12‐months showed consistent links with the affective components of depression in survivorship. Specifically, IFN‐γ and IL‐1β were robustly associated with greater consummatory anhedonia across follow‐up, and both markers—together with sTNFR1—were associated with poorer emotional functioning (greater negative affect). In addition, we observed time‐dependent associations for specific symptom dimensions: IL‐α was differentially related to motivational anhedonia across 12‐ and 24‐months, and sTNFR1 showed a small but significant interaction with time for consummatory anhedonia, even though no average association was present when effects were constrained to be constant over time. Notably, symptom course differed by domain, with consummatory anhedonia increasing from 12‐ to 24‐months whereas motivational anhedonia and negative affect remained relatively stable. Collectively, these results should be interpreted cautiously. Although several inflammatory biomarkers demonstrated statistically significant associations with specific depressive symptom domains, the exploratory design and lack of consistent associations across the complete biomarker panel preclude immediate clinical translation. Replication and mechanistic studies are required before inflammation‐guided interventions can be considered. While treatment modality was included as an adjustment variable, residual biological effects of previous chemotherapy or radiotherapy may still have influenced inflammatory marker concentrations beyond treatment completion. Finally, our analyses focused specifically on symptom domains rather than overall depression severity. Although this symptom‐oriented approach was central to the aims of the present study, future studies directly comparing domain‐specific symptom models with overall depression severity scores may help determine whether symptom‐specific analyses provide additional biological insight.

## 5 Conclusion

In conclusion, selected inflammatory biomarkers were associated with specific depressive symptom domains during colorectal cancer survivorship. However, these associations were not consistently observed across all inflammatory markers and should therefore be interpreted as exploratory. Future studies with repeated immune assessments, dedicated symptom measures, and independent replication cohorts are needed before firm conclusions regarding inflammation‐specific depressive symptom profiles can be drawn. Yet, our findings seem to suggest that inflammatory signaling might be associated with distinct depressive symptom dimensions in cancer survivorship. These results motivate future longitudinal studies with repeated immune and symptom assessments to clarify temporal ordering and to evaluate targeted anti‐inflammatory adjuncts to psychosocial care.

## Author Contributions

**Chris Hinnen:** conceptualization, data collection, writing – original draft, writing – review and editing. **Floortje Mols:** data collection, writing – review and editing. **Dounya Schoormans:** conceptualization, data collection, writing – review and editing.

## Funding

This study was supported with an Investment Subsidy Large (#91101002) of the Netherlands Organization for Scientific Research (The Hague, The Netherlands). This funding body had no role in the design of this study, data collection, analyses, interpretation, or manuscript writing.

## Ethics Statement

The PROCORE study was approved by the certified Medical Ethic Committee of Medical research Ethics Committees United (registration number: NL51119.060.14).

## Consent

Informed consent was obtained from all individual participants included in the study.

## Conflicts of Interest

The authors declare no conflicts of interest.

**Abbreviations**
CRC: Colorectal cancer
EF: Emotional Functioning
HADS: Hospital Anxiety and Depression Scale
HRQoL: Health‐related quality of life
IKNL: Netherlands Comprehensive Cancer Organisation
LMM: Linear Mixed Models
MFI: Multidimensional Fatigue Inventory
NCR: Netherlands Cancer Registry
PROFILES: Patient Reported Outcomes Following Initial treatment and Long term
SCQ: Self‐administered Comorbidity Questionnaire
SEP: Social Economic Position

## Data Availability

The data (including the blood samples themselves) will become freely available for non‐commercial scientific research, subject to study question, privacy and confidentiality restrictions, and registration (www.profilesregistry.nl).
